# A white matter-centered approach to investigate recurrence pathways in high-grade gliomas: a single-center retrospective study

**DOI:** 10.1007/s11060-025-05050-9

**Published:** 2025-06-03

**Authors:** Salvatore Marino, Grazia Menna, Francesco Doglietto, Davide Quaranta, Silvia Chiesa, Simona Gaudino, Rosellina Russo, Gianmaria Marziali, Marco Galeazzi, Quintino Giorgio D’Alessandris, Liverana Lauretti, Pier Paolo Mattogno, Rina Di Bonaventura, Naike Caraglia, Lucia Di Maio, Alessandro Olivi, Giuseppe Maria Della Pepa

**Affiliations:** 1 Neurosurgery Unit, Department of Neurosciences, Catholic University School of Medicine, Rome, Italy; 2 Department of Neurosurgery, Mater Olbia Hospital, Olbia, 07026 Italy; 3 Neurosurgery Unit, Department of Neurosciences, Fondazione Policlinico Universitario Agostino Gemelli, Istituto di Ricovero e Cura a Carattere Scientifico (IRCCS), Rome, Italy; 4 Neurology Unit, Department of Neurosciences, Fondazione Policlinico Universitario Agostino Gemelli, Istituto di Ricovero e Cura a Carattere Scientifico (IRCCS), Rome, Italy; 5 Department of Radiology, Radiotherapy and Hematology, Fondazione Policlinico Universitario A. Gemelli IRCCS, Rome, Italy; 6 Diagnostic Neuroradiology Unit, Fondazione Policlinico Universitario Agostino Gemelli, IRCCS, Largo A. Gemelli, 8, Rome, 00168 Italy; 7 Department of Neurosurgery, Fondazione Policlinico Universitario Agostino Gemelli, Largo Agostino Gemelli 1, Rome, 00168 Italy

**Keywords:** High grade glioma, Recurrence, White matter

## Abstract

**Background and aim:**

High-grade gliomas (HGGs) are aggressive primary brain tumors with inevitable recurrence. This single-center retrospective study investigates whether the anatomical proximity of HGGs to major white matter tracts influences progression and recurrence. The study explores the association between tumor location and recurrence type—local, remote, or ependymal—and whether recurrences align with adjacent white matter tracts.

**Methods:**

The study included patients with histopathologically confirmed recurrent HGGs who underwent reoperation. Primary tumors were categorized into four anatomical subgroups using a connectivity-based framework from the HCP 1065 Atlas: **Subgroup A**: Long Fronto-Temporo-Parietal Network **Subgroup B**: Temporal Pole Network (further divided into B1, B2, and B3 based on connectivity patterns) **Subgroup C**: Frontal Pole Network **Subgroup D**: Commissural and Projection Networks (further divided into D1 and D2). Recurrences were classified via post-contrast T1-weighted MRI as local, remote, ependymal. The Tract-to-Region Connectome (T-R-C) assessed the volumetric overlap between recurrence maps and main white matter bundles.

**Results:**

Of 41 patients, a significant correlation emerged between tumor subgroup and recurrence type (*p* = 0.0003). Subgroup A predominantly showed remote recurrences (68%), while B2, B3, C, and D2 had mainly local recurrences. Subgroup D1 had a predominance of ependymal recurrences (66.7%). Local and remote recurrences largely conformed to adjacent white matter distributions, with variations in timing of recurrence and survival observed across different groups.

**Conclusion:**

Our analysis, focused on exploring the spatial aspects of recurrence in relation to white matter anatomy, suggests that HGG recurrence patterns are strongly influenced by anatomical location and white matter architecture. Certain anatomical areas show a predisposition toward specific recurrence patterns. Recognizing these spatial dynamics may guide more precise surgical strategies, radiotherapy targeting, and recurrence risk assessment.

**Supplementary Information:**

The online version contains supplementary material available at 10.1007/s11060-025-05050-9.

## Introduction

High-grade gliomas (HGGs) are the most aggressive primary brain tumors, with inevitable recurrence despite advancements in treatment. While significant progress has been made in understanding their biology, the mechanisms driving tumor recurrence remain poorly understood [1–3]. Tumor microenvironments (TMEs) play a critical role in HGGs progression, and recent evidence suggests that myelin could act as a pro-differentiative niche - since tumor cell differentiation could be influenced by the selective upregulation of SOX10 - while glioma stem cells exploit Notch-Jagged1 signaling to migrate along demyelinated white matter tracts, potentially influencing both disease development and relapse mechanisms [4–13]. Despite extensive research on the molecular and cellular interactions between glioma cells and various brain structures at the microscopic level, their broader macroscopic implications remain largely unexplored, with the notable exception of the corpus callosum, which is the only well-established conduit for contralateral infiltration [14–16]. In particular, the correlation between tumor location, white matter connectivity, and recurrence patterns remains insufficiently explored.

Therefore, it may be helpful to improve the conventional understanding of tumor recurrence by adding a new dimension that connects the microscale processes in TMEs to the macroscale dynamics of recurrence patterns. This would reinforce the link between local biological mechanisms and large-scale anatomical behavior.

The primary aim of this single center retrospective study is to investigate whether the anatomical location of HGGs, particularly their proximity to major white matter tracts, influences tumor progression and recurrence. Specifically, it examines whether tumor location affects the type of recurrence (local, remote, or ependymal) and evaluates whether recurrences are spatially linked to, or propagate along, white matter tracts near the primary tumor.

## Methods

### Patient selection

We retrospectively identified patients with histopathologically confirmed recurrence of HGGs (according to WHO 2021 [17]) who underwent surgical resection at Fondazione Policlinico Universitario Agostino Gemelli between January 2021, and September 2024. Eligibility criteria included: (1) age over 18 years, (2) surgeries performed at our institution, (3) histologically confirmed HGGs at both initial surgery and follow-up, (4) gross total resection (GTR) confirmed within 48 h postoperatively using contrast-enhanced T1-weighted MRI reviewed by a radiologist, and (5) follow-up up to the second recurrence. Patients were excluded if they had incomplete data, were lost to follow-up, had histopathologically confirmed pseudoprogression or radionecrosis [18], presented with multifocal or multicentric gliomas, or if recurrence was not operated or histopathologically confirmed.

The study was specifically designed to include only patients with histopathologically confirmed recurrence of HGG, effectively focusing on cases that underwent reoperation. This decision enhances the homogeneity and reliability of the analyzed results while minimizing potential confounding factors; however, it inherently reduces the sample size.

For each patient, we collected demographic, clinical, and imaging data, including age, anatomical location of the primary lesion, time to recurrence, and location of the recurrent lesion.

The Institutional Review Board (IRB) of Fondazione Policlinico Gemelli IRCCS approved this study (ID 7028).

### Tumor categorization based on anatomical location

Primary tumors were categorized using a connectivity-based framework derived from the HCP 1065 Atlas. The subdivision was designed to adopt a functional anatomical approach, grouping regions that, even if anatomically distant, participate in the same anatomo-functional systems, while separating regions that, despite their anatomical proximity, serve different anatomo-functional systems.

Primary tumors were classified into the following areas:

#### Area A

(Long Fronto-Temporo-Parietal Network) identified as the territory of the superior longitudinal fasciculus (SLF) and arcuate fasciculus (AF). This region encompasses the supramarginal gyrus (SMG), angular gyrus (AG), posterior segments of the superior, middle, and inferior temporal gyri (STG, MTG, ITG), and the frontal operculum.

#### Area B

(Temporal pole network) comprises brain regions with bundles converging at the temporal pole and further subdivided into: B1, including the ventro-lateral surface of the temporal pole with the sagittal stratum, corresponding to the ILF distribution; B2 including the mesial temporal pole, Limen Insulae, and orbito-frontal gyri, representing the UF territories; B3 including the medial temporal surface of the temporal lobe (uncus, parahippocampal gyrus), which is associated with the Amygdala-Hippocampal formation.

#### Area C

(Frontal pole network) includes brain regions from two interwoven systems—the cingulate Bundle and forceps Minor—that converge at the mesial frontal pole. Consequently, this area encompasses the frontal pole, subcallosal area, cingulate gyrus and genus of corpus callosum.

#### Area D

(Commissural and Projection networks) includes diffuse regions, representing territories where commissural and projection fiber systems are prevalent. Further subdivisions within Area D include D1, which encompasses the Superior Frontal Gyrus (SFG), Middle Frontal Gyrus (MFG), Superior Parietal Lobe (SPL), Precuneus (PrC), and medial occipital gyri (m-OG), aligned with the callosal radiation; and D2, which includes the Central Lobe (CL) and Paracentral Lobe (PCL), areas associated with the Cortico-Spinal Tract (CST).

### Recurrence classification and analysis

Recurrences were classified on follow-up post-contrast T1-weighted MRI into three primary types based on their spatial relationship to the surgical cavity and ventricular ependyma: (1) Local – in contact with the prior surgical cavity but not the ventricular ependyma; (2) Remote – confined to the white matter, with no contact with the surgical cavity or ependyma; and (3) Ependymal – showing diffusion along the ventricular ependyma.

Local recurrences were further classified as “contiguous” if they overlapped with white matter tracts related to the group or “non-contiguous” if they did not. Remote recurrences were classified as “consonant” if they arose along the expected distribution of white matter bundles within the group or “dissonant” if they occurred outside these distributions.

The Tract-To-Region Connectome (T-R-C) was calculated using DSI Studio to measure the volumetric overlap between the Recurrence Distribution Map and main white matter bundles. This provides a detailed spatial relationship analysis between recurrences and the brain’s white matter architecture.

### Statistical analysis

We conducted a Chi-Square Test for Association to assess the relationship between tumor subgroups and first recurrence type (Ependymal, Local, Remote). Fisher’s Exact Test was applied to ensure robustness.

Kaplan-Meier survival analysis (KM) was performed to evaluate overall survival (OS) and time to recurrence, with analyses stratified by tumor subgroup and recurrence type. Log-Rank and Wilcoxon tests were used to compare survival distributions, with statistical significance set at *p* < 0.05.

All analyses were conducted using JMP Pro 17.0 (SAS Institute Inc., Cary, NC, USA).

## Results

### Patient characteristics

A total of 41 patients met the inclusion criteria. All patients were IDH-wt. Moreover, 22 were MGMT methylated, and 9 were not; the methylation profile was not available in 10 cases. All patients underwent postoperative STUPP protocol. Detailed data for each patient are provided in Supplementary Table 1.

### Recurrence patterns and distribution maps analysis

A significant relationship was observed between tumor subgroup and recurrence type (Fisher’s Exact Test, *p* = 0.0003). Specifically, Subgroup A was the only one strongly associated with remote recurrences (68%), while Subgroups B2, B3, C, and D2 exhibited almost only local recurrences. Subgroup D1 exhibited a predominance of ependymal recurrences (66.7%). Subgroup B1 exhibited mixed results. (Table 1).

**Table 1:** Summary of the main characteristics of the subgroups

Group	n° Patients	n° Recurrences	Total local		Total remote		Epend.	Mean recurrence time (months)			OS (months)		
			Contig.	Non-Contig.	Consonant	Dissonant		Local	Remote	Ependym	Local	Remote	Ependym
A	12	18	6 (33.3%)		11 (61.11%)		1 (5.5%)	24.9			42.35		
			5 (27.7%)	1* (5.5%)	11 (61.11%)	-		14	19.5	14	21	32.77	18
B1	7	8	-		2 (25%)		6 (75%)	16.4			31.1		
					1 (12.5%)	1** (12.5%)		-	14	17.4			32,4
B2	4	4	4 (100%)		-		-	15.2			35,1		
			4 (100%)	-				15.2	-	-	35,1		
B3	2	3	3 (100%)		-		-	12.6					
			3 (100%)	-				12.6	-	-			
C	6	6	6 (100%)		-		-	10.2			18.5		
			6 (100%)	-				11	-	-	18.5		
D1	6	6	2 (33.33%)		-		4 (66.66%)	11.5			18,6		
			2 (33.33%)	-				15		9.75	22		16,9
D2	4	4	3		1		-	24			34.5		
			3	-	1	-		24	8		34.5		

Summary of the main clinical characteristics across subgroups. For each group, the total number of patients and total number of recurrences are reported, followed by the distribution of recurrence types: local (contiguous or non-contiguous), remote (consonant or dissonant), and ependymal. Percentages are calculated based on the total number of recurrences per group. Mean recurrence time is expressed in months and is stratified by recurrence location (local, remote, or ependymal). Overall survival (OS), also expressed in months, is reported similarly. Asterisks (*) indicate specific subtypes or notable cases referenced in the main text. [*Non contigous Local recurrence originating at the superior margin of the surgical cavity, located in the inferior parietal lobe, and extending toward the superior parietal lobe: ** Remote recurrence recurred from the right temporal lobe to the left frontal lobe]

All but one (in Group A) Local recurrences were contiguous, and all but one (in Group B1) Remote recurrences were consonant. Moreover, Visual inspection of Distribution Maps and T-R-C revealed a strong overlap between recurrences and the main White Matter Bundles associated with each Subgroup (Table 2).

**Table 2:** Tract-to-Region connectome relative to different groups

White matter bundle	T-*R*-Connectome*
GROUP A	
AF/SLF	L:0.362434; R:0.216261
IFOF	L: 0.040741; R: 0.049859
ILF	L: 0.034921; R: 0.092784
CC	L: 0.004233; R: 0.018182
CST	L: 0.000000; R: 0.000000
GROUP B-2	
UF	L: 0.238479
IFOF	L: 0.118568
ILF	L: 0.070694
GROUP C	
CB	L: 0.162389
Fm	L: 0.116513
UF	L: 0.002347
AF	L: 0.018106

Tract-to-Region Connectome (T-R-C) profiles for each subgroup, representing the relative involvement of major white matter bundles in tumor connectivity. The values denote normalized connection weights between the tumor regions and specific tracts in the left (L) and right (R) hemispheres. Subgroup A was primarily connected with fronto-parietal associative tracts (AF/SLF, IFOF, ILF); B2 with ventral limbic pathways (UF, IFOF, ILF); and C with medial structures (CB, Fm). Only subgroups with ≥ 3 unilateral recurrences are shown. These T-R-C patterns strongly overlapped with the recurrence locations observed on distribution maps, suggesting a topographic correlation between recurrence type and tumor-tract connectivit. These connectivity patterns closely mirrored the recurrence distributions seen in the anatomical maps, suggesting a topographic relationship between recurrence type and tumor-tract anatomy. However, it is important to consider the limitations of this method. Tumor growth often distorts surrounding brain structures, and when tumor segmentations are registered to an undistorted MNI template, some degree of misalignment is inevitable. This mismatch—exacerbated by necrotic or cystic changes—can affect the perceived overlap between tumors and tracts. To address this, the analysis emphasizes relative, rather than absolute, tract involvement. By focusing on the tracts showing the highest relative overlap for each subgroup, this approach provides insight into the most probable anatomical pathways of recurrence while accounting for the distortive effects of tumor growth

### Group A (long fronto-temporo-parietal network)

**Fig. 1:**
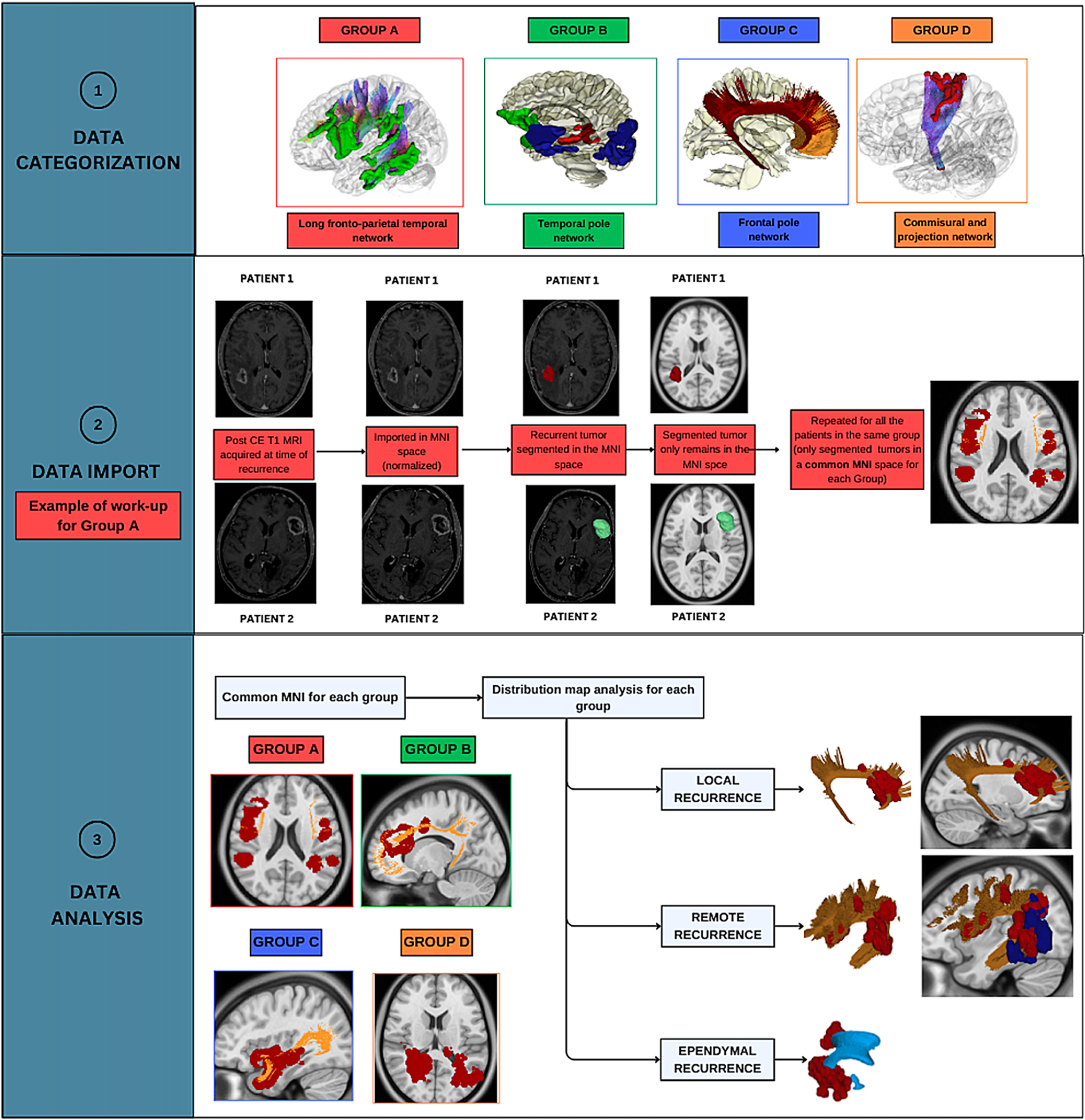
**Data Categorization**: Patients were classified into different groups based on the location of the primary tumor, as detailed in the text. Group A: Tumors located along the territory of the SLF/AF complex. Group B: Tumors located along the territory of the ILF and Sagittal Stratum (B1), Uncinate Fasciculus (B2), and Hippocampal Formation (B3). Group C: Tumors located in the territory of the Cingulate Bundle and Forceps Minor. Group D: Tumors located in the territory of the Commissural pathways (mainly callosal radiation, D1) and Projection pathways (mainly the corticospinal tract, D2). **Data Import**: For each patient, the T1-MDC scan acquired at the time of radiological diagnosis of recurrence was normalized to the Montreal Neurological Institute (MNI) space using the integrated normalization software in DSI Studio (DSI Studio, http://dsi-studio.labsolver.org). The registration quality was visually inspected in all cases, with manual adjustments performed when necessary. Recurrences were segmented from the original MRI and aligned to the MNI space. Recurrences from patients within the same subgroup were sequentially imported into a common MNI space. This process resulted in a “Distribution Map” for each group, integrating all recurrences of patients belonging to the same group within the MNI space. The primary white matter tracts associated with each subgroup (e.g., AF/SLF for Group A) were generated using the ICBM 152 Atlas template on the MNI space with Distribution Map. Each recurrence was first assessed individually and classified as either “contiguous” or “non-contiguous” for local recurrences and “consonant” or “dissonant” for remote recurrences. After individual classification, recurrences were assessed collectively, and volumetric overlap between each subgroup’s Distribution Map and the corresponding white matter bundles was visually inspected and quantified using the Tract-to-Region Connectome function in DSI Studio. Separate Distribution Maps were generated for local and remote recurrences, as well as for ependymal recurrences within each group. Distribution maps containing fewer than two recurrences per side were excluded from the analysis, even if depicted

**Fig. 2:**
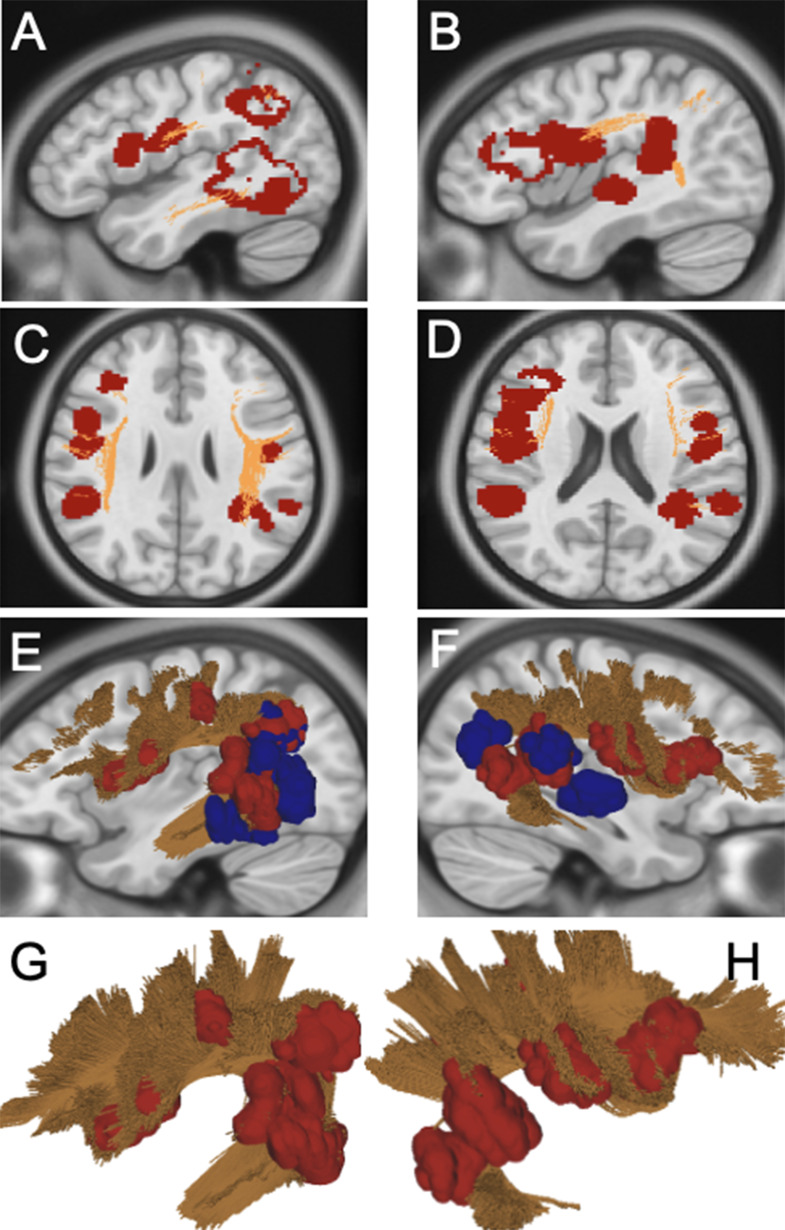
(**A**–**D**) Sagittal and axial 2D scan showing Distribution Map of Group A. In red are highlighted the recurrences belonging to this group segmented into the MNI space. Specifically, local recurrences arose in all cases from the antero-inferior aspect of the surgical cavity toward the temporal termination of the AF. Regarding remote recurrences, two occurred at the level of the posterior temporal lobe from a primitive located at the SMG/AG; four at the level of the SMG/AG from a primitive located to the posterior temporal lobe; five at the level of fronto-parietal operculum, namely pars triangularis, pars opercularis and subcentral gyrus from a primitive located at the SMG/AG. (**E-F**, **G-H**) 3D rendering of the recurrences in the left and right hemispheres. In red, recurrences, in blu original tumor, in yellow AF/SLF

In this group, 12 patients experienced 18 recurrences, with remote recurrences being the most frequent (62%), followed by local (33%) and ependymal (5%) events. Both local and remote recurrences were strongly aligned with the SLF and AF, with a T-R-C analysis confirming 2–10 times higher connectivity for these tracts compared to unrelated ones (Figs. 1, 2). Examples of Group A recurrences are provided in Supplementary Fig. 2.

### Group B (temporal pole network)

**Fig. 3:**
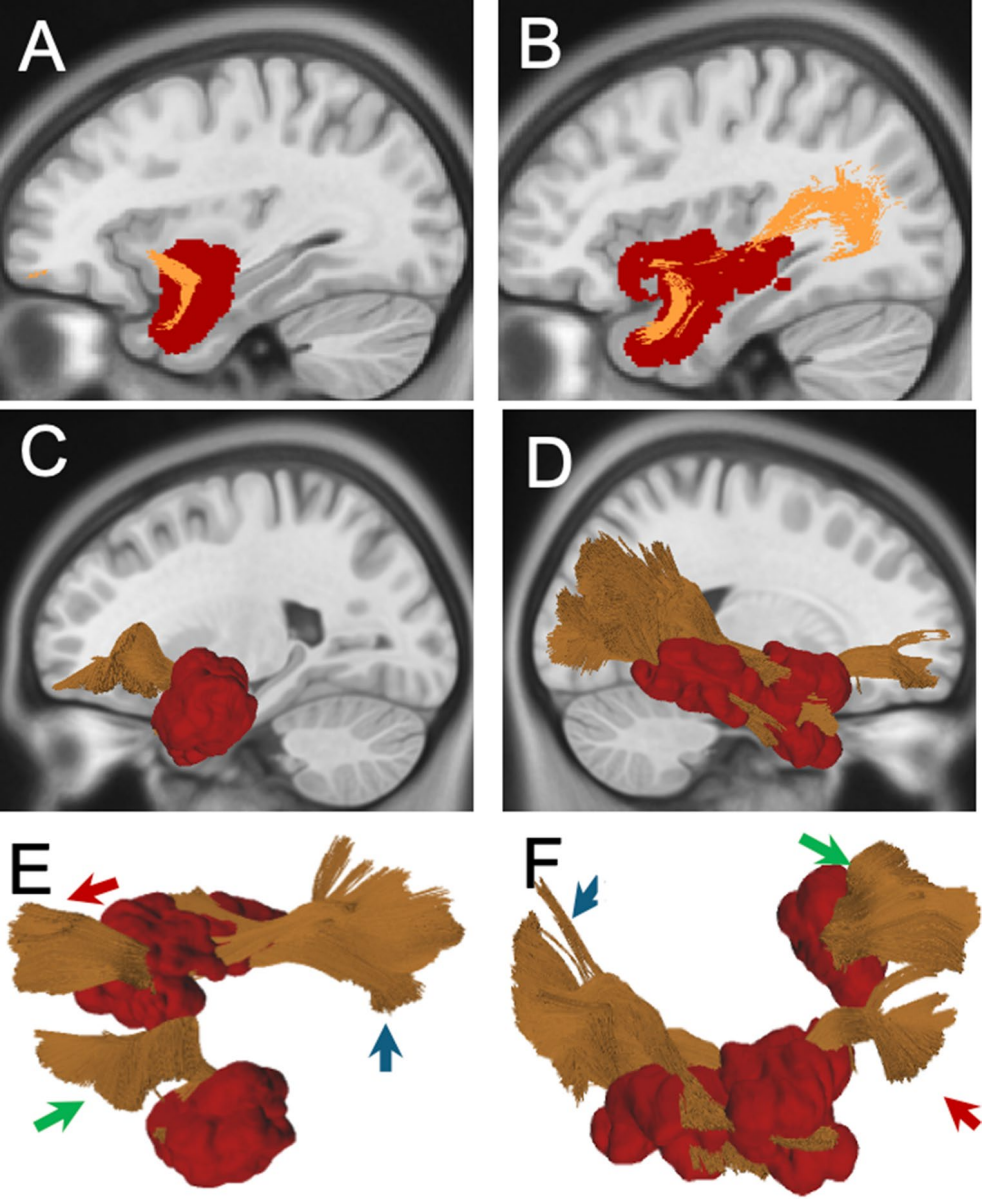
(**A-B**) 2D distribution map of Group B. Sagittal scan of the left side is presented on the left, with the recurrences segmented into the MNI space (in red) and UF in orange. Sagittal scan of the right side is presented on the right, with the recurrences segmented into the MNI space (in red) and UF and ILF in orange. (**C-D**) 3D rendering of the Group B distribution map: On the left, the anatomical relationship between recurrences and the uncinate fasciculus (UF) in the left hemisphere is shown. On the right, the anatomical relationships are illustrated between recurrences, and the UF/ILF in the right hemisphere. (**E-F**) 3D-rendering of the left uncinate fasciculus (green arrow), the right uncinate fasciculus (red arrow), and the right inferior longitudinal fasciculus (blue arrow) and recurrences (in red)

Comprising 13 patients with 15 total recurrences, this group exhibited 8 local, 5 ependymal, and 2 remote recurrences. Ependymal recurrences were prominent in Subgroup B1, where tumors displayed direct ventricular contact. Subgroup B2 demonstrated exclusively local recurrences, aligned with the UF, as suggested by visual inspection and by T-R-C (Fig. 3). Subgroup B3 showed recurrences along the HF and fimbria. Examples of Group B recurrences are provided in Supplementary Fig. 3.

### Group C (frontal pole network)

**Fig. 4:**
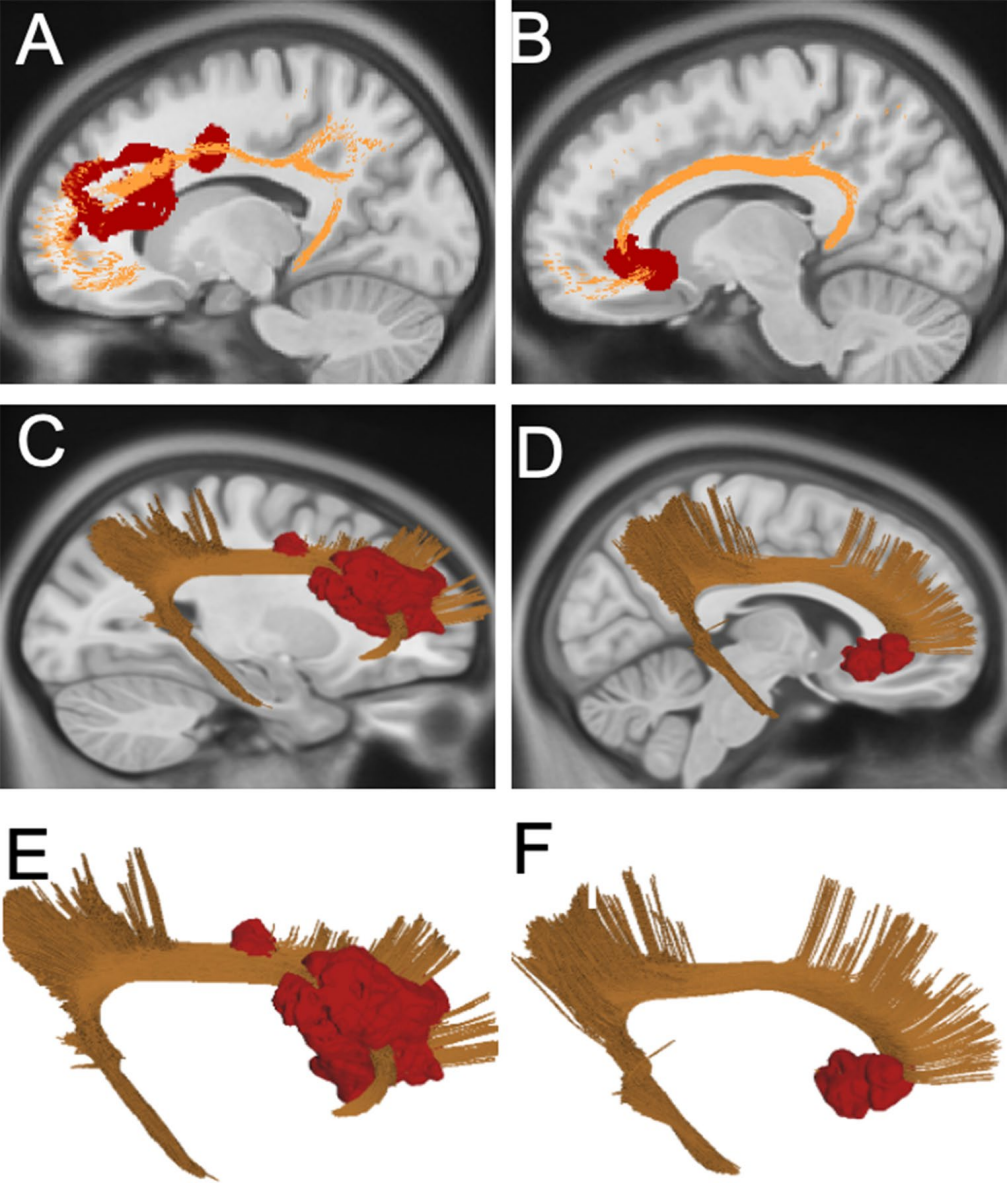
(**A-B**) 2D Distribution map of Group C. Sagittal scan of the left side is presented on the left, with the recurrences segmented into the MNI space (in red) and CB in orange. Sagittal scan of the right side is presented on the right, with the recurrences segmented into the MNI space (in red) and CB in orange. In all cases, recurrences were local, arose from the posterior margin of the surgical cavity and progressed along the cingulate gyrus. In four out of six cases, the recurrences also involved the GunuCC; (**C**–**F**) 3D rendering of the Group C distribution map: On the left, the anatomical relationship between recurrence and Cingulate Bundle in the left hemisphere is shown. On the right, the anatomical relationships are illustrated between recurrences, and the Cingulate Bundle in the right hemisphere

All six patients in this group had local recurrences. These recurrences extended along the CG, involving the genus of the CC in four cases. Distribution maps highlighted a strong association with the cingulate bundle and forceps minor, with T-T-R Connectome significantly higher for this structure compared to other nearby tracts (Fig. 4). Examples of Group C recurrences are provided in Supplementary Fig. 4.

### Group D (commissural & projection networks)

**Fig. 5:**
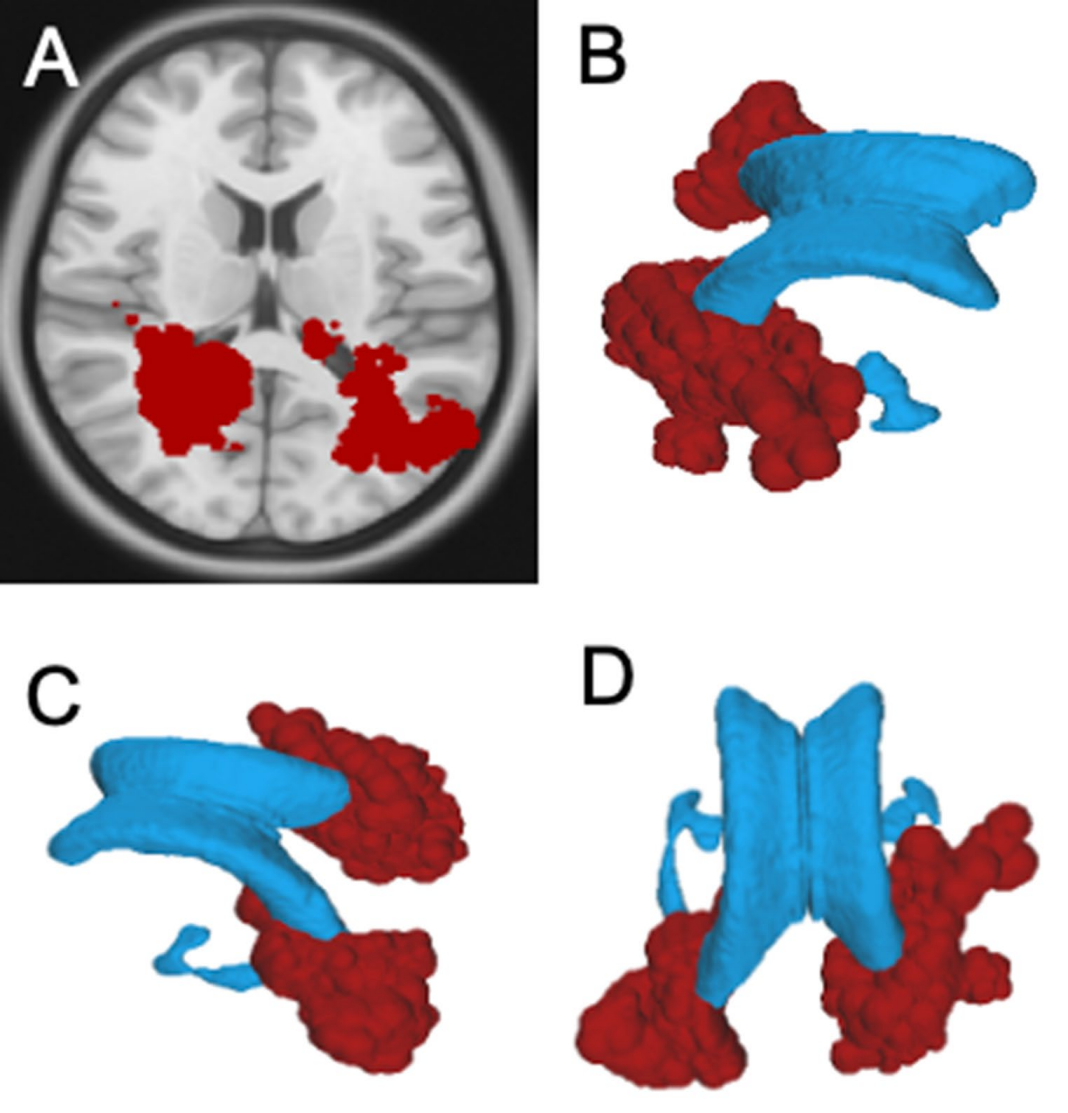
2D Distribution Map of ependymal recurrences. (**A**) Axial scan is presented, with the recurrences segmented (**B**–**D**) into the MNI space in red. 3D-rendered map illustrating the distribution of ependymal recurrences, with recurrences shown in red and their anatomical relationship to the lateral ventricles highlighted in blue. Regardless of location, the main characteristic associated with an ependymal route of spread was a “ventricolocentric” shape of the tumor at onset, marked by an enhancing nodule directed toward, or in direct contact with the sub-ependymal layer at onset and FLAIR alteration extending toward the ventricle. The primary ventricular walls involved in tumor recurrences were the atrium and temporal horn

Among 10 patients, recurrences varied by subgroup. In subgroup D1, two patients presented with primary enhancing lesions in the MFG, each developing local recurrences toward the body of the CC. Four patients had primary enhancing lesions at the level of the occipital lobes with a direct contact with the ventricular ependyma at onset, and developed ependymal recurrences, with eventually further subependymal seeding (Fig. 5).

Within Subgroup D2, two patients presented with lesions in the central lobe and developed local recurrences extending toward the centrum semiovale. Two other patients exhibited primary lesions in the paracentral gyrus, and the first developed a remote recurrence in the splenium of the CC, while the second case recurred locally toward the splenium of the CC.

Examples of Ependymal recurrences are provided in Supplementary Fig. 5.

### PFS and OS analysis

**Fig. 6:**
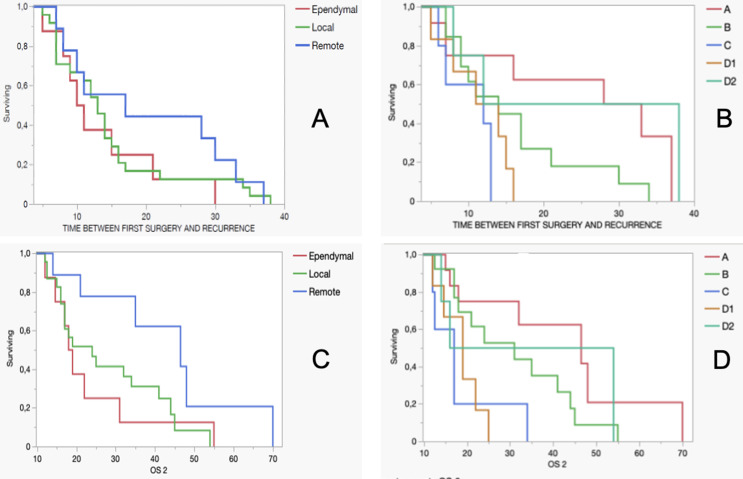
(**A**) Kaplan-Meier analysis of time to first recurrence across recurrence types showed no statistically significant differences. The Log-Rank Test yielded χ² = 1.466 (*p* = 0.481), and the Wilcoxon Test produced χ² = 1.336 (*p* = 0.513). While descriptive trends suggest earlier recurrence in ependymal cases, these differences were not statistically significant. (**B**) Kaplan-Meier analysis of time to first recurrence across tumor subgroups revealed a statistically significant difference based on the Log-Rank Test (χ² = 11.449, *p* = 0.022). However, the Wilcoxon Test did not confirm this significance (χ² = 5.392, *p* = 0.249). (**C**) Kaplan-Meier analysis of overall survival across recurrence types indicated a trend toward statistical significance, though it did not meet conventional thresholds. The Log-Rank Test yielded χ² = 4.610 (*p* = 0.100), while the Wilcoxon Test produced χ² = 4.519 (*p* = 0.104). (**D**) Kaplan-Meier analysis of overall survival across tumor subgroups demonstrated statistically significant differences. The Log-Rank Test resulted in χ² = 14.081 (*p* = 0.007), and the Wilcoxon Test yielded χ² = 10.773 (*p* = 0.029)

Across all subgroups, the median time to recurrence was 10.5 months for ependymal recurrences, 13 months for local recurrences, and 17 months for remote recurrences. Although descriptive data suggest that remote recurrences occur later compared to local and ependymal types, these differences were not statistically significant (Fig. 6).

When considering recurrence timing across tumor groups, Group A exhibited the longest median time to recurrence at 30.5 months, while Groups C and D1 had the shortest times at 12 and 12.5 months, respectively. Group B had a median recurrence time of 14 months, and Group D2 showed an intermediate recurrence time of 25 months. Despite these variations, a statistically significant difference in recurrence timing across groups was observed (Fig. 6).

Regarding overall survival (OS), the median and mean OS were 18.5 and 23.6 months for patients with ependymal recurrences, 24 and 27.9 months for local recurrences, and 46.5 and 43.5 months for remote recurrences. Although these differences indicated a trend toward significance, they did not consistently reach statistical significance (*p* < 0.05).

Regardless of recurrence type, Group A demonstrated the longest survival, with a median and mean OS of 46.5 and 42.4 months, respectively. This was followed by Group B with 31 and 30.8 months. In contrast, Groups C and D1 had the shortest survival times, with median and mean OS of 17 and 18.5 months for Group C, and 18.6 months for Group D1. Group D2 showed intermediate survival outcomes with median and mean OS of 35 and 34.5 months, respectively. These findings indicate that tumor group significantly impacts OS (*p* = 0.0070), with patients in Groups C and D1 experiencing the poorest survival outcomes.

## Discussion

This study highlights the central role of white matter tracts in shaping HGG recurrence patterns, reinforcing the need to move beyond the traditional lobar anatomy framework. By examining how recurrence pathways align with white matter tracts, the findings demonstrate that tumor behavior is influenced not only by the anatomical location of the primary lesion but also by the structural and functional connectivity of adjacent white matter bundles. This white matter-centered perspective offers a new lens for understanding the spatial and temporal dynamics of HGG recurrence [19].

Although limited in number, other studies in the literature have investigated the tendency of gliomas to grow along white matter tracts [20–24]. However, unlike the present study, most of these focus on the natural history of gliomas, primarily low-grade ones, and are centered on individual white matter tracts (e.g., the Corpus Callosum and the Uncinate Fasciculus). In contrast, our study specifically examines recurrences of glioblastomas following surgery and considers all major white matter tracts. By doing so, it provides a comprehensive perspective on the potential influence that white matter networks may exert on tumor recurrence.

### Recurrence pattern: local, remote, ependymal

Our findings suggest a strong association between tumor location at onset and the type of recurrence. Results align with previous findings in the literature, which report that up to 90% of HGG recurrences occur near the primary tumor site [2, 3]. In our series, most of the cases exhibit pure local recurrences (51%; 65% after excluding ependymal recurrences).

However, our findings highlight the need for a more nuanced analysis to understand recurrence patterns fully. For instance, Group A demonstrated a statistically significant tendency toward remote recurrences rather than local ones. Notably, remote recurrences comprised 67% of all recurrences in this group and exhibited longer recurrence time and OS compared to other groups. In contrast, Groups B and C exhibited predominantly local recurrences with shorter mean recurrence time and OS compared to remote recurrences of Group A. These observations underscore the importance of considering white matter connectivity when evaluating glioblastoma recurrence, as different tracts not only shape recurrence pathways but could also influence the timeline of disease progression. The reasons behind these differences remain unclear, although intrinsic architectural features of white matter—such as fiber density, diameter, and myelin thickness—may play a role [20–25].

In this study, 24% of cases exhibited recurrence through an Ependymal diffusion, a proportion notably lower than the 50–60% ependymal involvement reported in previous studies [26]. This discrepancy is likely attributable to the stringent inclusion criteria of our cohort, which exclude rapidly progressing cases or those ineligible for surgical re-intervention. Interestingly, the majority (90%) of tumors that developed ependymal recurrences in this study originated in the temporal and occipital lobes bilaterally.

Although the literature frequently associates ependymal invasion with rapidly progressing HGGs and poorer clinical outcomes [27], our findings revealed notable differences depending on tumor location. Ependymal recurrences from temporal tumor at onset were associated with longer mean recurrence intervals when compared to Ependymal recurrences from occipital tumor onset, which in turn exhibited the shortest mean recurrence times and OS of all subgroups. However, the intrinsic nature of our analysis—primarily aimed at investigating spatial aspects of recurrence—and the limited sample size do not allow for robust conclusions regarding recurrence timing and survival outcomes.

The distinct characteristic of Ependymal recurrences was their “ventricolocentric” morphology at onset, despite anatomical location (Supp. 5). Molecular investigations are warranted to determine if molecular factors influence this recurrence pattern [27–29].

These findings are consistent with those of Akeret et al. [30], who proposed a glioma grading system based on the anatomical relationship between the tumor and the ventricular/periventricular structures. Tumors in extensive contact with the ventricular system or adjacent periventricular regions were associated with poorer prognosis. One could speculate that, in an early stages, some glioblastomas remain in more peripheral regions and recur along white matter tracts, while in later phases they may progress toward the ventricles. However, certain glioblastomas exhibit ventricular spread from the onset, suggesting the existence of distinct subtypes with different patterns of spatial progression. Naturally, these remain speculative hypotheses that will need to be validated in future studies.

### Distribution maps analysis

Distribution Maps analysis revealed nuanced patterns of recurrence. In particular, Group A recurrences strongly overlap with the distribution of SLF/AF both for local and remote recurrences, and bilaterally. Group B2 demonstrates a recurrence pattern that appears to correspond closely to the trajectory of the UF. Group B3 typically exhibits a recurrence pattern overlapping HF and fimbria. Group C recurrences follow a precise white matter-based pathway, primarily along the CB itself and to a lesser extent along the Fm. Group D (especially Frontal and Parietal components) recurrences display a tendency to recur predominantly via the Callosal Radiation and, possibly through the Corona Radiata. Group B1 and Group D1 (Occipital Component) recurrences have a tendency not to follow regional white matter bundles but rather an Ependymal pattern.

Even within one region, tumor precise origin affects recurrence patterns and timing. For example, in Group B, all tumors originate in the temporal pole, yet their exact location defines three recurrence patterns: B1, B2, and B3.

### Clinical relevance

Overall, our results emphasize the need for a conceptual shift in neuro-oncology, moving away from the traditional lobar anatomy framework to one focused on White Matter. Functional anatomy and cerebral connectivity are not merely passive structures within which glioblastomas develop but active contributors that influence tumor behavior, including the recurrence type, location, and timing.

These findings highlight the necessity for personalized treatment strategies that consider not only molecular characteristics of the tumor but also its anatomical location. Understanding potential recurrence pathways can inform surgical planning, radiotherapy targeting, and surveillance strategies.

Therefore, creating a “map of high-risk areas” would facilitate a more focused treatment approach, allowing for targeted interventions in regions with a higher likelihood of recurrence while preserving unaffected areas. This strategy paves the way for a personalized, patient-tailored approach.

Even if such an approach has already been enquired in radiotherapy planning [1, 31–33], the possibility to tailor surgical resection is a distant perspective, being hindered by overwhelming issues such as the precise orientation within the surgical field, brains shift and intraoperative discrimination among different white matter bundles.

Furthermore, this study emphasizes a fundamental aspect directly affecting how cerebral anatomy is interpreted in neuro-oncology: it underscores the need for a conceptual shift from traditional lobar anatomy to one focused on white matter when observing and interpreting the development, spread, and recurrence of gliomas. Furthermore, these findings underscore the potential of developing recurrence prediction maps based on white matter connectivity, which could guide postoperative surveillance and improve early detection of recurrences.

### Limitations

Despite the promising insights offered by this study, several limitations must be acknowledged. The small sample size restricts the generalizability of the findings, thus limiting the potential for deeper statistical analyses. Only patients with histopathologically confirmed recurrence of HGG were included in the study. This choice ensures greater homogeneity and reliability in the analyzed results while minimizing potential confounding factors. However, it significantly reduces the number of analyzed cases and introduces an inherent bias by including only patients eligible for a second surgery on the residual tumor, effectively excluding those not suitable for reoperation. Indeed, the focus on surgically amenable cases may have omitted patients with more aggressive or multifocal tumor growth, which may not follow the same patterns observed in this patient cohort. Additionally, the analysis was restricted to the first or second recurrence, leaving the later stages of glioblastoma progression unexplored.

This study is presented as a pilot investigation, highlighting the need for larger, multicenter studies with extended follow-up periods to validate and expand upon these findings.

## Conclusion

The findings of this study suggest that HGG recurrence patterns and timing are closely tied to the tumor’s anatomical origin, with specific brain regions exhibiting distinct pathways of spread that align with white matter tracts. Certain anatomical areas show a predisposition toward specific recurrence types (local, remote, ependymal), suggesting that the underlying white matter architecture actively shapes tumor behavior. By recognizing the role of brain connectivity in influencing recurrence type and location, this paradigm shift could inform the development of more precise therapeutic strategies, such as personalized surgical planning, radiotherapy targeting, and recurrence risk mapping.

## Electronic supplementary material

Below is the link to the electronic supplementary material.

Supplementary Material 1

## Data Availability

No datasets were generated or analysed during the current study.
