# Supplementary material for: A white matter-centered approach to investigate recurrence pathways in high-grade gliomas: a single-center retrospective study
Source: J Neurooncol. 2025 Jun 3;174(1):177–90. doi: 10.1007/s11060-025-05050-9 (PMC12198263; doi:10.1007/s11060-025-05050-9)
Supplement: Supplementary file 1 — Supplementary Material 1 [file 11060_2025_5050_MOESM1_ESM.docx]

**Supplementary Table 1**- Main characteristics for each patient.

| **AGE** | **Methylation** | **GROUP** | **SUBGROUP** | **1ST ENHANCING NODULE** | **FLAIR ALTERATION** | **VENTRICULOCENTRIC** | **I° RECURRENCE** | **II° RECURRENCE** | **PFS** | **OS** |
| --- | --- | --- | --- | --- | --- | --- | --- | --- | --- | --- |
| 65 | UM | A |  | right posterior T3 | sagittal stratum | **N** | L |  | 16 | 32 |
| 47 | / | A |  | left posterior T2 and T3 | posterior part AF | N | L | R | 5 | 16 |
| 58 | UM | A |  | right SMG | SLF/AF | N | R |  | 33 | 70 |
| 66 | M | A |  | left SMG | SLF/AF | N | R | R | 37 | 48 |
| 45 | M | A |  | left AG | T1 and extC | N | L | R | 7 | 18 |
| 44 | M | A |  | left SMG | AF | N | L | R | 7 | 15 |
| 59 | M | A |  | right posterior T1 and AG | - | Y | L | E | 14 | 18 |
| 59 | M | A |  | right AG | AF | N | R | R | 28 | 47,5 |
| 57 | M | A |  | left posterio T3 | - | N | R |  | 10 | 21 |
| 64 | \ | A |  | posterior left T1-T2 | - | N | L |  | 35 | 41 |
| 53 | UM | A |  | posterior left T2 | - | N | R |  | 30 | 36 |
| 62 | M | A |  | right posterior T1 | AF | N | R |  | 7 | 24 |
| 67 | \ | B | B1 | right ventro-lateral temporal pole | sagittal strat | Y | R |  | 17 | 21 |
| 56 | M | B | B1 | right ventro-lateral temporal pole | Sagitt strat/AF | NA | E |  | 9 | 17 |
| 44 | M | B | B1 | Right ventral temporal pole | around lesion | NA | R |  | 11 | 35 |
| 52 | M | B | B1 | right ventral temporal pole | around lesion | NA | E |  | 30 | 55 |
| 69 | M | B | B1 | ventro-lateral left temporal pole | sagittal stratum | NA | L | E | 17 | 41 |
| 63 | UM | B | B1 | fusiform and mid-temporal | - | Y | E |  | 10 | 18 |
| 70 | M | B | B1 | Fusiform gyrus and mid-temporal | - | Y | E |  | 21 | 31 |
| 55 | M | B | B3 | right mesial temporal lobe | temporal pole, uncus and prahyppocampus | N | L |  | 14 | 24 |
| 53 | UM | B | B3 | right mesial temporal lobe | temporal pole, uncus and prahyppocampus | N | L |  | 7 | 45 |
| 62 | M | B | B2 | left fronto-temporal operculum and Limen | fronto-temporal | N | L |  | 7 | 17 |
| 50 | M | B | B2 | left superior temporal pole sx | superio temporal pole and limen | N | L |  | 11 | 22 |
| 41 | \ | B | B2 | left superio temporal lobe, limen and orbito-frontal | fronto-temporal and limen | N | L |  | 34 | 44 |
| 60 | M | B | B2 | right superior temporal pole. | superior temporal pole and limen | N | L |  | 9 | 12,5 |
| 66 | \ | C |  | Left lateral frontal pole | frontal pole, cingulum, CC | N | L |  | 15 | NA |
| 49 | \ | C |  | left mesial haemorrhagic frontal pole | - | N | L |  | 13 | 17 |
| 51 | UM | C |  | right frontal pole and subcallosal area | - | N | L |  | 7 | 12.5 |
| 52 | \ | C |  | left frontal pole and genu of CC | around tumor | N | L |  | 6 | 12 |
| 73 | UM | C |  | Left cingulate gyrus | around tumor | N | L |  | 13 | 34 |
| 52 | M | C |  | Left subcallosal area and genu of CC | frontal pole, cingulum, CC | N | L |  | 12 | 17 |
| 71 | M | D | D1 | Right MFG | - | N | L |  | 14 | 19 |
| 21 | M | D | D1 | Left MFG | - | N | L |  | 16 | 25 |
| 58 | UM | D | D1 | left occipital gyri | toward ventricle | Y | E |  | 5 | 19 |
| 52 | M | D | D1 | left occipital gyru | toward ventricle | N | E |  | 11 | 22 |
| 41 | UM | D | D1 | left medial occipital lobe | toward ventricle | Y | E |  | 8 | 12 |
| 45 | M | D | D1 | medial right occipital lobe | - | Y | E |  | 15 | 14.6 |
| 36 | M | D | D2 | Left Subcentral gyrus |  | N | L |  | 38 | 54 |
| 54 | \ | D | D2 | Right hand knob |  | N | L |  | 22 | 34 |
| 69 | M | D | D2 | left paracentral lobe | toward ventricle | N | R |  | 8 | 14 |
| 54 | \ | D | D2 | right paracentral lobule | - | N | L |  | 12 | 16 |


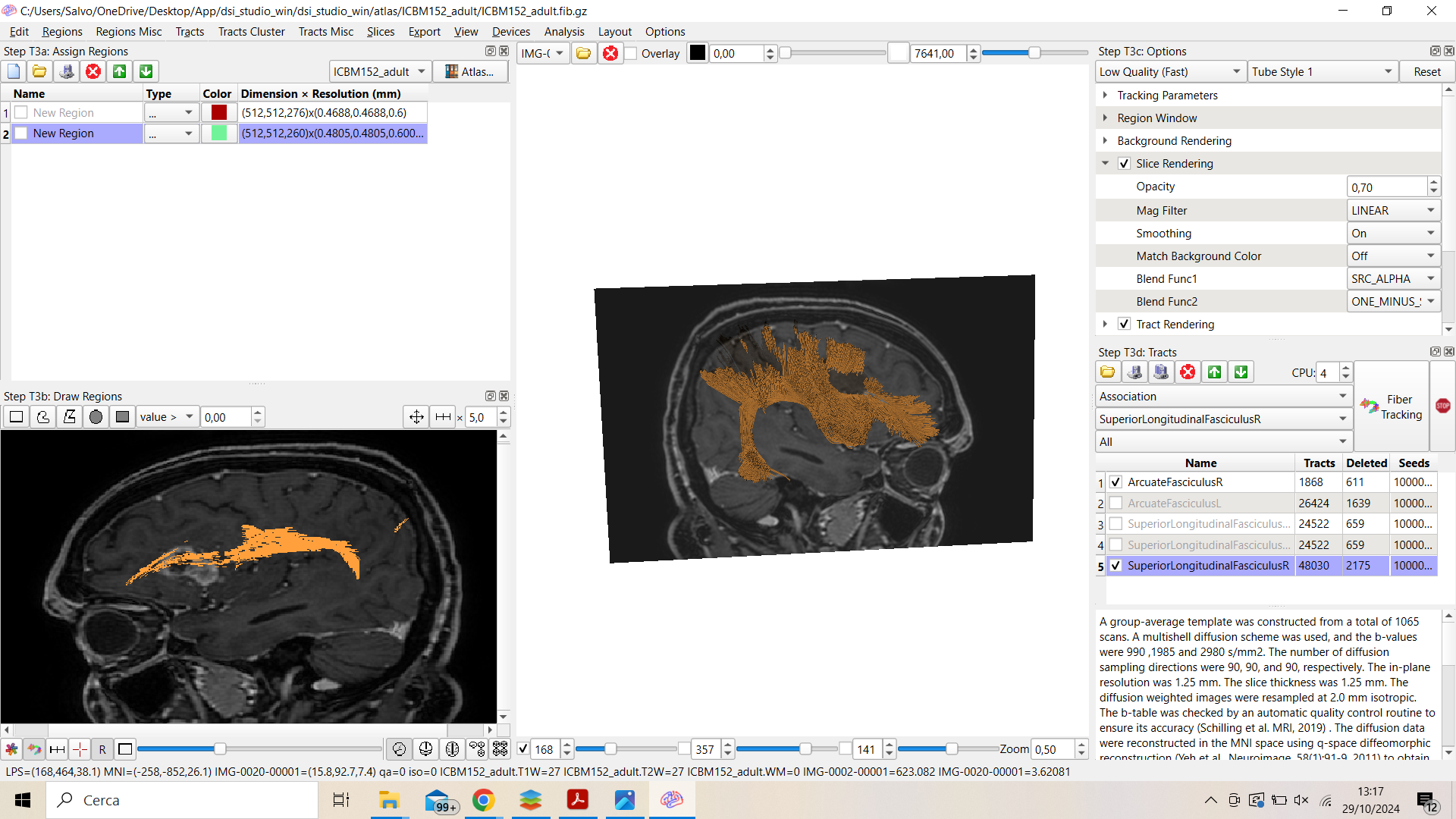

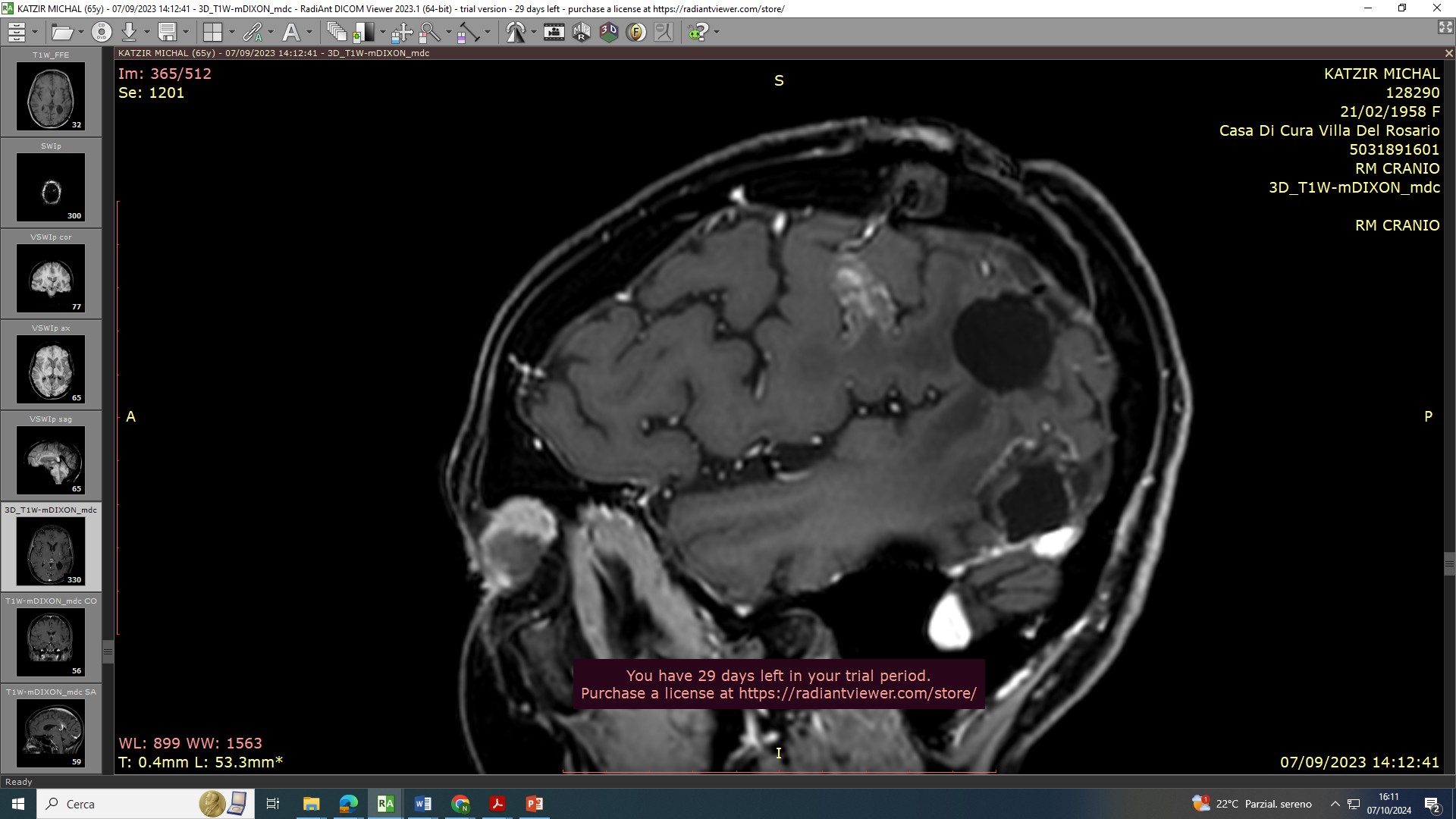

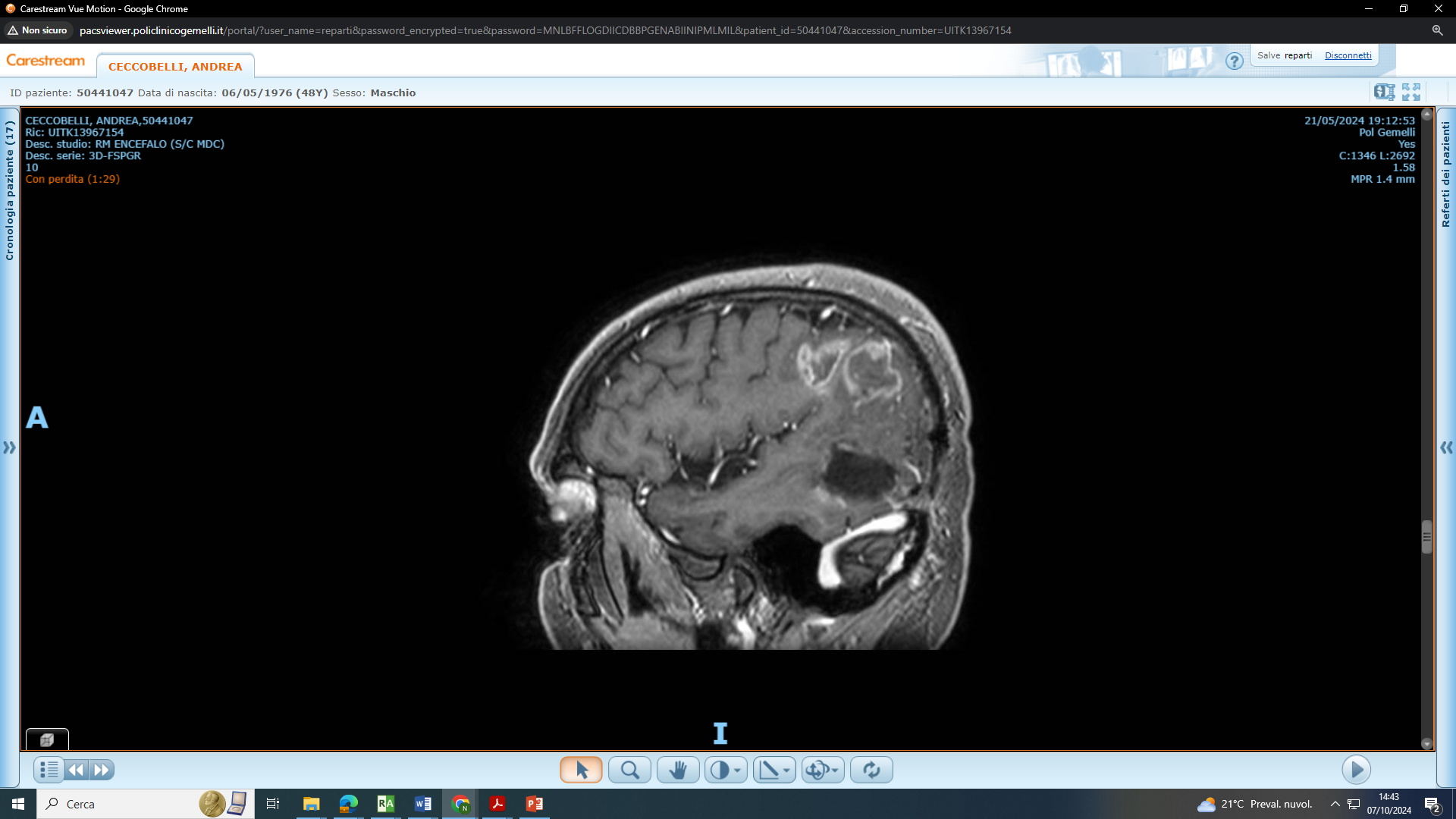

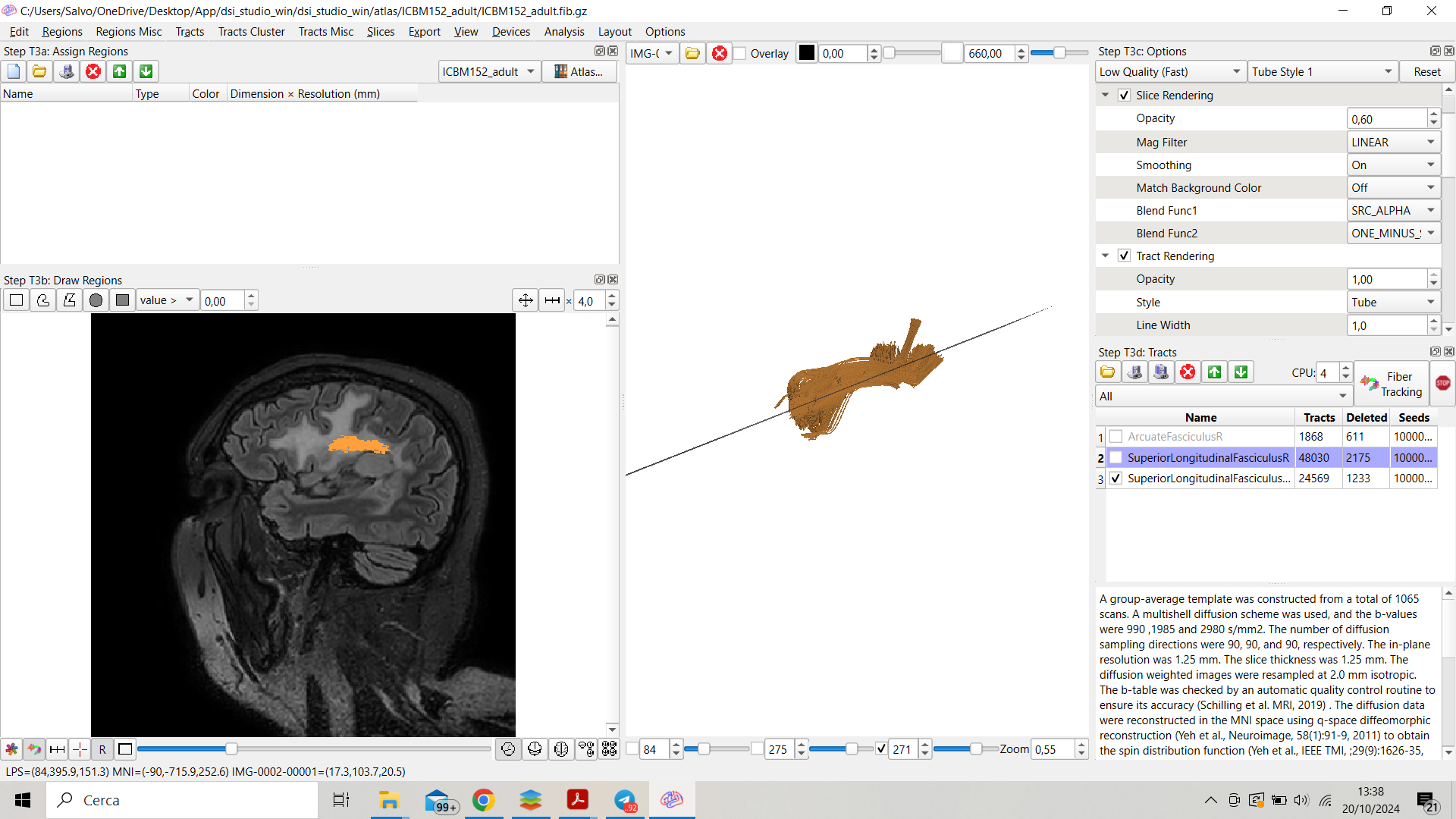

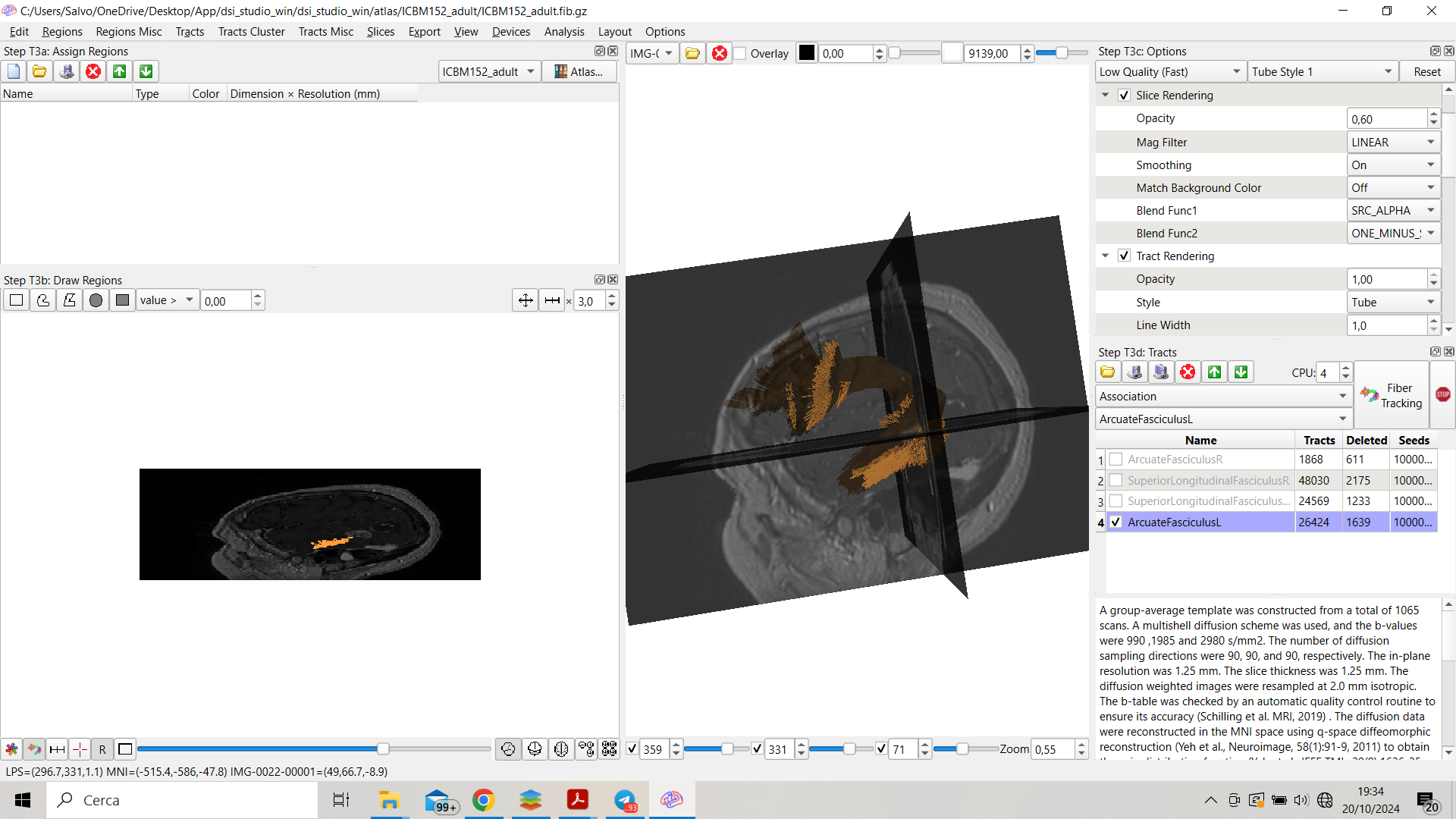

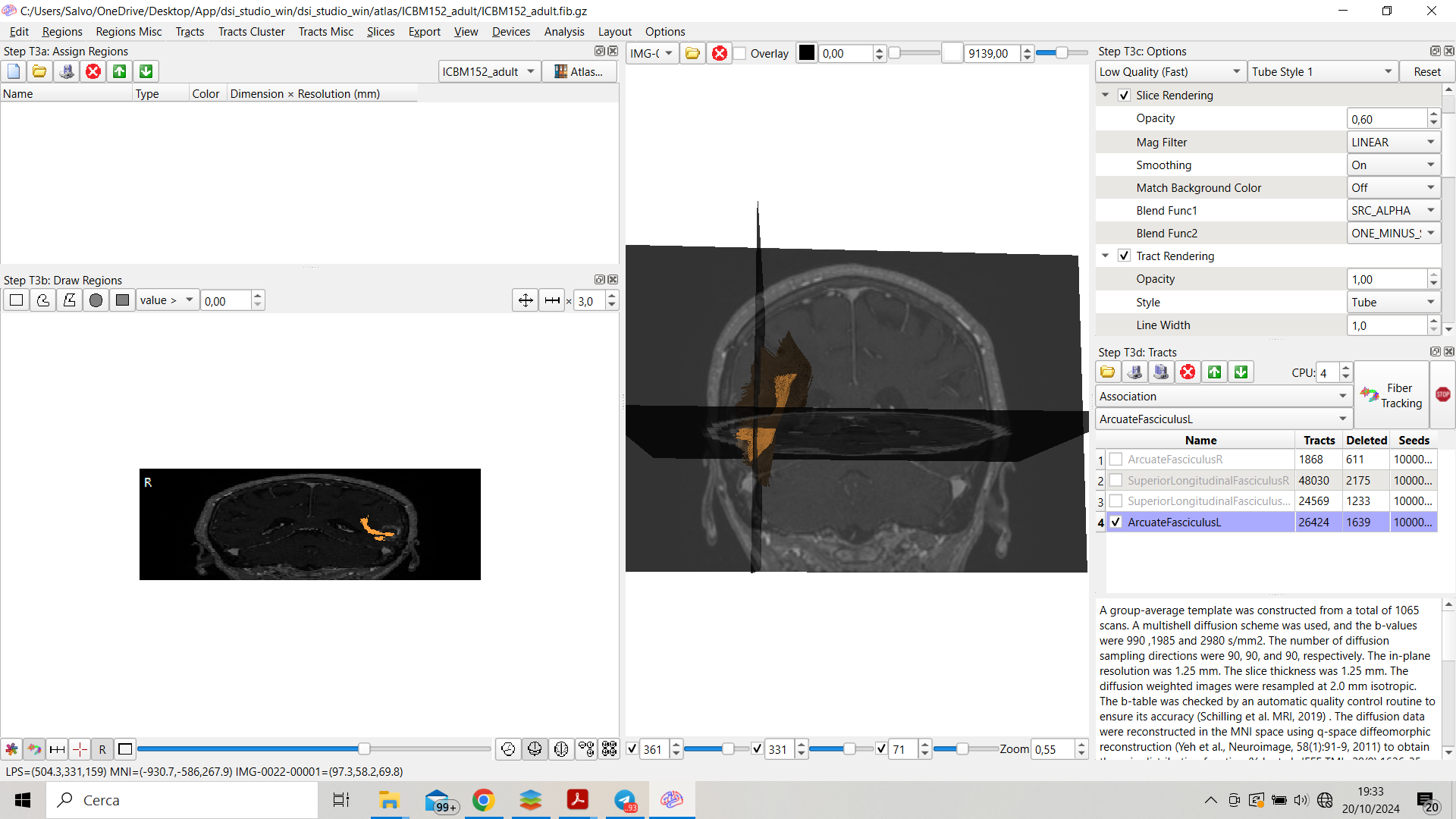


**Supplementary Figure 2**. A) Sagittal T1-MDC scan showing the surgical cavity at the posterior third of the temporal lobe and a remote recurrence at the inferior parietal lobe (IPL) level. B) Sagittal T1-MDC scan showing the first surgical cavity at the posterior third of the temporal lobe, the surgical cavity of a remote recurrence at the IPL level, and an incoming recurrence at the ventral Rolandic region. C) Sagittal T1-MDC scan showing a remote recurrence at the frontal operculum and its relationship with the AF (the primary lesion was deep to the IPL and is not visible in this scan). D) Sagittal FLAIR scan showing the first surgical cavity (blue arrow) at the parieto-temporal junction, the remote recurrence (red arrow) at the frontal operculum, and its relationship with the SLF. Notably, the image demonstrates FLAIR alterations overlaying the SLF/AF territory. E-F) Sagittal and coronal T1-MDC scans showing the previous surgical cavity, local recurrence, and the relationship between the local recurrence and the temporal component of the AF.


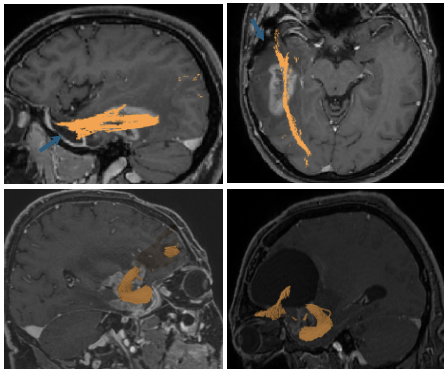


**Supplementary Figure 3**. A-B) Sagittal and axial T1-MDC scans showing a remote recurrence along the sagittal stratum, the primary tumor's surgical cavity located at the ipsilateral temporal pole (blue arrow), and its relationship with the ILF, depicted in orange. C) Sagittal T1-MDC scan showing a local recurrence originating from a primary tumor located at the mesial temporal pole. Notably, the relationship between the UF (in orange) and the local recurrence is highlighted. D) Sagittal T1-MDC scan showing a local recurrence arising from a primary tumor located in the orbitofrontal region. The image highlights the anatomical relationship between the UF and the local recurrence.


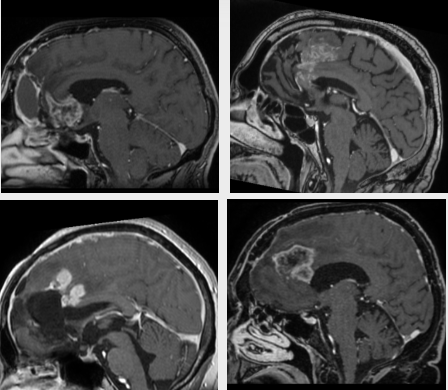


**Supplementary Figure 4**. (A) Local recurrence in the subcallosal area, extending toward the cingulum. The primary surgical cavity is visible in the frontal pole. (B) Local recurrence involving the cingulate gyrus and genu of the corpus callosum. The primary tumor was located in the frontal lobe and is not visible in this slice. (C) Local recurrence affecting the cingulate gyrus, partially extending to the medial surface of the superior frontal gyrus (SFG) and the genu of the corpus callosum. The primary surgical cavity is visible in the mesial frontal lobe. (D) Recurrence involving the cingulate gyrus and genu of the corpus callosum. The primary tumor was located in the frontal pole region and is not visible in this slice.


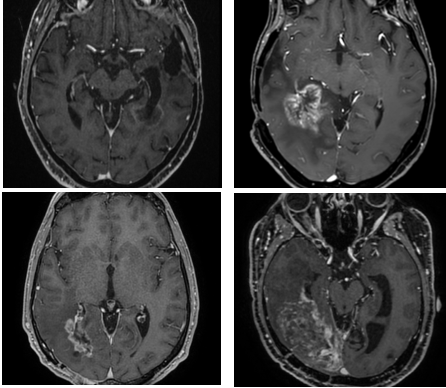


**Supplementary Figure 5** (A) Ependymal recurrence along the wall of the atrium of the left lateral ventricle, originating from a primary tumor in the left temporal pole. (B) Ependymal recurrence along the wall of the atrium of the right lateral ventricle, originating from a primary tumor in the right temporal pole. (C-D) Ependymal recurrence along the wall of the atrium of the right lateral ventricle, originating from a primary tumor in the right occipital pole.
